# Editorial: Towards a psychophysiological approach in physical activity, exercise, and sports, volume II

**DOI:** 10.3389/fpsyg.2024.1434218

**Published:** 2024-06-26

**Authors:** Pedro Forte, José Eduardo Teixeira, Daniel Leite Portella, Diogo Monteiro

**Affiliations:** ^1^CI-ISCE, Higher Institute of Educational Sciences of the Douro, Penafiel, Portugal; ^2^Research Center in Sports, Health, and Human Development, Covilhã, Portugal; ^3^Department of Sports Sciences, Instituto Politécnico de Bragança, Bragança, Portugal; ^4^Research Center for Active Living and Wellbeing (Livewell), Instituto Politécnico de Bragança, Bragança, Portugal; ^5^Department of Sports Sciences, Polytechnic Institute of Guarda, Guarda, Portugal; ^6^Research Center in Sports Performance, Innovation and Technology (SPRINT) Recreation, Guarda, Portugal; ^7^Master Program in Innovation in Higher Education, University of São Caetano do Sul, São Caetano do Sul, Brazil; ^8^Department of Human Kinetics, School of Education and Social Sciences (ESECS), Polytechnic of Leiria, Leiria, Portugal

**Keywords:** exercise, sports, performance, psychophysiological, physical activity

The critical role of physical activity in fostering physical and mental health and wellbeing has been well-documented across diverse populations and contexts. Recent studies published in leading journals provide compelling evidence of the multifaceted benefits of exercise, from improving physical metrics such as body mass index (BMI) and waist circumference to enhancing mental health and subjective wellbeing. These insights underscore the importance of promoting physical activity as a cornerstone of public health. Health related physical activity was a very important discussed topic during COVID-19 pandemic, and for that reason, it is cleared illuminated the critical intersection between physical activity and mental health. That's in line with the published study in this Research Topic by Tomezzoli et al. on young Italian athletes. The authors revealed the detrimental effects of sports activity limitations on mental health. The enforced inactivity during lockdowns led to increased anxiety, depression, and a sense of loss among children, adolescents, and young adults. This allows to remind the psychological benefits of regular physical activity and the need for robust policies to ensure continued access to sports and recreational activities. However, where and when to do is a critical aspect and environment may play an important role on wellbeing. The study from Sirotiak et al. highlight significant differences in how physical activity influences psychological wellbeing depending on the environment. This research underscores the importance of considering geographic and environmental factors when promoting physical activity, ensuring that interventions are tailored to the specific needs and contexts of different populations.

The society, culture, and wellbeing were also part of the discussion in this Research Topic. The study from Jacinto et al. delves into the effects of exercise on individuals with intellectual and developmental disabilities. The review highlighted significant reductions in BMI and waist circumference, underscoring the potential of tailored exercise programs to improve physical health metrics in this population. This study was particularly crucial to understand the barriers to participating in physical activities, which can exacerbate health disparities. The study from Liao et al. identified perceived health, social support, and self-esteem as key mediating factors, reinforcing the idea that the benefits of physical activity extend beyond physical health to encompass mental and social dimensions. The transcultural analysis by Liang et al. on sports science students in France and China allowed to understand how cultural contexts shape values and attitudes toward physical activity. Finally, Liu et al. explored how partnership dynamics in DanceSport, suggesting that the strong interpersonal relationships and engagement can significantly boost performance, pointing to the broader psychosocial benefits of sports and dance activities.

Regarding to improving the sports performance, four studies measured performance, perceptions, competence, and strategies for long-term and to end the careers. Della Tommasina et al. explored the preventive benefits of exercise, examined a dry-land strengthening program for swimmers. The findings showed that exercises using elastic bands could significantly reduce shoulder injuries, highlighting the importance of injury prevention strategies in sports training. This approach not only enhances athletic performance but also ensures the long-term health and participation of athletes. Other study by Portella et al. on Brazilian football goalkeepers highlights the developmental differences in strength and force variables across age groups. This study provided critical data for age-appropriate training regimens, which are essential for the optimal development of young athletes. The impact of professional certification on perceptions of expertise and credibility among golf instructors was investigated by Yang et al.. In their study the perceived quality of instruction can significantly influence learning outcomes and participation intentions, emphasizing the need for professional development in sports education. The study from Teixeira et al. developed an interview guide for the retired Portuguese football players. This instrument allows to assess the long-term impacts of sports careers on quality of life. Finally, the Chen et al. study focused on systematic desensitization training to reduce competitive anxiety in Latin dance athletes. This approach demonstrates how psychological interventions can complement physical training to enhance overall athletic performance. Altogether, several psychological variables played an important role in physiological performance, reinforcing the importance to better understand the concept of Psychophysiology.

Altogether, these studies collectively highlight the indispensable role of physical activity, exercise, and sports in enhancing various dimensions of health and wellbeing. They also stress the importance of continued research and tailored interventions to address specific needs across different populations. As we advance, integrating these insights into public health strategies will be key to fostering a healthier, more active society. This Research Topic aimed to offer valuable insights into the complex relationship between psychological factors, cognition, and physiological variables within the domains of physical activity, exercise, and sports. By exploring these multifaceted interactions through a comprehensive psychophysiological approach, we have achieved a deeper understanding of new strategies for optimizing performance, promoting health, and improving the overall quality of life in sports and exercise contexts. However, benefits of physical activity help to offer a holistic approach to health and wellbeing. It is important to seek further studies on the psychophysiological concept to continue uncovering strategies for enhancing health and performance in diverse populations.

## Author contributions

PF: Writing – original draft. JT: Writing – review & editing. DP: Writing – review & editing. DM: Writing – review & editing.

